# *Cryptococcus neoformans* Strains and Infection in Apparently Immunocompetent Patients, China

**DOI:** 10.3201/eid1405.071312

**Published:** 2008-05

**Authors:** Jianghan Chen, Ashok Varma, Mara R. Diaz, Anastasia P. Litvintseva, Kurt K. Wollenberg, Kyung J. Kwon-Chung

**Affiliations:** * Shanghai Changzheng Hospital, Shanghai, People’s Republic of China; † National Institutes of Health, Bethesda, Maryland, USA; ‡ University of Miami, Miami, Florida, USA; § Duke University Medical Center, Durham, North Carolina, USA

**Keywords:** Cryptococcosis, China, AIDS, Cryptococcus neoformans, C. gattii, molecular strain type, immunocompetent host, research

## Abstract

To determine the population structure of the cryptococcosis agents in China, we analyzed the genotype of 120 *Cryptococcus neoformans* and 9 *Cryptococcus gattii* strains isolated from 1980 through 2006 from cryptococcosis patients residing in 16 provinces of mainland China. A total of 71% (91/129) of the clinical strains isolated from 1985 through 2006 were from patients without any apparent risk factors. Only 8.5% (11/129) were from AIDS patients; the remaining 20.5% (27/129) were from patients with underlying diseases other than HIV infection. One hundred twenty of the 129 isolates were *C. neoformans* serotype A, mating type *MAT*α strains that exhibited an identical M13-based VNI subtype, which was distinguishable from the reference VNI molecular type. The 9 remaining isolates were serotype B, *MAT*α strains of *C. gattii* and portrayed a typical VGI molecular type. Data analyzed from multilocus sequences showed no variation and that these Chinese *C. neoformans* isolates belong to a cluster that has phylogenetically diverged from the VNI reference strain. Our finding that most cryptococcosis patients in China had no apparent risk factor is in stark contrast with reports from other countries.

## Abstract 2

High incidence of cryptococcosis in these patients is in contrast to reports from other countries.

Cryptococcosis is caused by 2 species in the genus *Cryptococcus*, *C. neoformans* and *C. gattii* (*1*). *C. neoformans* (serotypes A, D, and AD) is found worldwide and causes cryptococcosis most frequently in AIDS patients (*2*,*3*). *C. gattii* (serotypes B and C) is geographically restricted and is infrequently diagnosed in AIDS patients except in some areas of Africa (*4*). The most widely used approaches for cryptococcal strain typing include the following: PCR fingerprint analysis based on microsatellite DNA using M13 primers (*5*,*6*) and (GACA)_4_ repeats (*7*), amplified fragment length polymorphisms (*8*), sequence analysis of the rDNA intergenic spacer regions I and II (IGSI and IGSII) (*9*), and multilocus gene sequence typing (MLST) (*10*). These molecular approaches showed genetically distinguishable subgroups for each serotype. For example, phylogenetic analysis of the IGS1-5.8S rDNA-IGSII sequence showed 3 genotypes, 1a, 1b, and 1c, among the strains of serotype A collected worldwide (*9*). Likewise, PCR fingerprint patterns based on M13 microsatellite DNA identified 2 major genotypes among the strains of serotype A. In this group, the VNI strain type was the most common genotype found worldwide (*5*). Furthermore, MLST showed 3 genetic subpopulations among strains of serotype A as well as subpopulations unique to certain geographic areas as was the case with strains from Botswana (VNB) in Africa (*10*). A *C. neoformans* genotype unique to Botswana showed epidemiologic importance of strain type. Rare genotypes of *C. gattii* have been reported from the recent outbreak of cryptococcosis on Vancouver Island in Canada (*11*). Finding *C. gattii* strains of a rare genotype in geographic areas outside of subtropical and tropical zones (*12*) further underscores the epidemiologic importance of this rare genotype.

In spite of extensive global strain typing, isolates from the People’s Republic of China have rarely been included (*5*,*8*,*9*). Recently, data released by the Chinese Department of Health indicated that by October 2006, the total number of HIV patients and those identified as AIDS patients was 183,733 and 40,667, respectively. (www.chinacdc.net.cn/n272442/n272530/n294176/n340510/15099.html)

From 1999 through 2006, a total of 224 articles on cryptococcosis have been published in Chinese journals; 2,850 case-patients were included. We cited 6 representative reports from these papers (*13*–*18*). In these cases, only 69 strains (0.2 %) were associated with AIDS. The population structure of *C. neoformans* in China, however, has not been fully explored. In this study, we have used various molecular typing approaches to analyze the population structure of the cryptococcosis agent in China. 

## Materials and Methods

### Cryptococcal Isolates

A total of 129 strains isolated from patients included 120 *C. neoformans* and 9 *C. gattii* (Appendix Table). The identities of all strains were confirmed according to routine diagnostic tests (*2*). L-canavanine-glycine-bromothymol blue medium was used to differentiate the 2 species (*19*). We used a set of strains representing the known molecular types within the 2 species as a reference: WM 148 (serotype A, VNI), WM 626 (serotype A, VNII), Bt 63 (serotype A, Botswana), WM 628 (serotype D, VNIII), WM629 (serotype AD, VNIV), WM 179 (serotype B, VGI), WM 178 (serotype B, VGII), WM 161 (serotype B, VGIII) and WM 779 (serotype C, VGIV)(6). The strain H99 (serotype A) was also included (*20*). The isolates were stored in 25% glycerol at –80^o^C until use and were maintained on yeast peptone dextrose (1% yeast extract, 2% peptone, 2% glucose) agar at 25^o^C during this study.

### Serotype and Mating Type

The CryptoCheck kit (Iatron, Tokyo, Japan) was used for serotyping. Mating type was determined by crossing all the *C. neoformans* strains with B-3501 (*MATα*) and JEC20 (*MATa*) and *C. gattii* strains with NIH112 (*MATα*) and NIH198 (*MATa*) on V-8 agar (*11*,*21*). A few strains of *C. neoformans* that showed ambiguous mating results were analyzed by PCR using serotype A *MATα* and *MATa* allele specific primers of either the *STE12* or *STE20* gene (*22*).Yeast cultures were grown overnight in yeast extract glucose broth (0.5% yeast extract, 2% glucose) at 30°C. Genomic DNA was extracted from all strains as previously described (*23*).

### Genotyping

Genotyping approaches included PCR and DNA sequencing. PCR fingerprint analysis was based on M13 primers for microsatellite DNA as well as primers containing sequence repeats of (GACA)_4_ and *URA5*–restriction fragment length polymorphism (RFLP). Sequencing was performed for partial IGSI-5.8S-IGSII region of rDNA cluster, and MLST involving 8 genes located on 7 different chromosomes (*10*). PCR fingerprints using either the microsatellite-specific primer M13 (5′-GAGGGTGGCGGTTCT-3′) (*5*) or the (GACA)_4_ sequence repeats (*7*) were generated as described. The *URA5-*RFLP profiles were generated after digestion of the PCR fragment containing the *URA5* gene sequence with *Sau*961 and *Hha*1 (*6*). IGSI sequence analysis of the LrDNA gene was derived from PCR amplicon products (≈1.7 kb) generated by the primer pair combination LR11 (5′-TTACCACAGGGATAACTGGC-3′) and 5SR (5′-GGATCGGACGGGGCAGGGTGC-3′) (*9*). The amplification reactions were carried out in microtubes at a final volume of 50 μL. The PCR mix contained 50–100 ng of genomic DNA, 0.5 mmol/L of the forward and reverse primer pairs, 1.0 U DyNAzyme II polymerase, 1.5 mmol/L MgCl_2,_ and 200 mmol/L of each dNTP. The reaction was performed in an MJ Research PTC-100 thermocycler (GMI, Inc., Ramsey, MN, USA) and consisted of a denaturation step at 94°C for 1 min, followed by 40 cycles: 2 min of denaturation at 95°C, 1 min of annealing at 57°C, and 3 min of extension at 72°C. A final elongation step was conducted at 72°C for 7 min. The amplicon product was purified with QIAquick PCR Purification Kit (QIAGEN, Valencia, CA, USA). Amplicon synthesis was confirmed by agarose gel electrophoresis. Sequencing reactions used the forward primer IGS1F (5′-CAG ACGACTTGAATG-GGAACG-3′), located at positions 3613–3633 of the LrRNA region and the reverse primer IG2R (5′-ATG CAT AGA AAG CTG TTG G-3′), located at position 791 of the IGS1 region. Sequencing reactions were carried out with an ABI 3730 capillary sequencer (Applied Biosystems, Foster City, CA, USA) and followed the protocol described by Diaz et al. (*9*). Sequencing alignments of the IGS region were executed with MegAlign (DNASTAR, Inc., Madison, WI, USA) and visually corrected. Phylogenetic tree construction used PAUP* version 4.0 (*24*) with parsimony analysis (heuristic search, stepwise addition, random addition sequence, nearest neighbor interchange, 100 maximum tree). Reliability of the character was checked by using bootstrap analysis with 500 replications.

### Construction of Dendrogram Based on M13 RFLP

To construct a dendrogram based on M13-generated DNA fingerprints, 12 strains representing the different provinces of China were chosen, along with 6 reference strains. The PCR product of the reference strain VNI was used twice in the panel (adjacent to the size marker and in the last lane). A total of 15 major bands that ranged in size from 3,054 bp to 506 bp were identified across the lanes. Data were coded as 1 and 0; 1 represented the presence of a band and 0, its absence. This data matrix was coded into NEXUS format for input into the program PAUP* (*24*). PAUP* was used to generate a maximum parsimony phylogeny using a heuristic search algorithm. For the heuristic search, 10 random-addition replicates were performed. The starting tree for each replicate was obtained by stepwise addition. Seven replicates returned the same most parsimonious tree, which had a tree score of 18 steps. To quantify clade robustness, 500 nonparametric bootstrap replicates were performed. Bootstrap percentages >50% are indicated above the branches of the maximum parsimony phylogenetic tree. The tree figure for the maximum parsimony analysis was created using FigTree version 1.0 (available from http://tree.bio.ed.ac.uk/software/figtree).

### MLST

For MLST analysis, DNA fragments of 8 unlinked genes that included *CAP10, GPD, IGS1, LAC1, PLB1, SOD1, TEF1,* and *URE1,* were amplified by PCR (*10*) from 6 randomly chosen *C. neoformans* strains from China. DNA sequencing was carried out by using the dideoxy method, and sequences were compared with previously published sequences from the global collection of serotype A strains (*10*). To visualize the genetic relationships among different MLST genotypes, sequences were automatically aligned by using Sequencher 4.1 (Gene Codes Corp., Ann Arbor, MI, USA); the alignment was imported into MacClade 4.05 and edited manually. Because of the observed incongruence in the genealogies of several genes, combined sequence data for all 88 worldwide isolates (*10*) were analyzed with the neighbor-joining method using uncorrected (“p”) genetic distances. Statistical support for the phylogenetic groups was assessed by bootstrap analysis using 1,000 replicate data sets.

### Virulence Study

Three Chinese strains of *C. neoformans*, CHC114, CHC186, and CHC193, were compared for their virulence in mice with 3 serotype A reference strains, H99, WM148 (VNI), and WM626 (VNII). Ten 9-week old female BALB/c mice were infected intranasally with 5 × 10^7^ cells of each strain (*23*) and were monitored for survival.

## Results

The conditions of most patients (81.3%) were diagnosed at Shanghai Changzheng Hospital; the remaining patients (18.6%) were diagnosed at other hospitals. Patients without any recognizable predisposing factor for cryptococcosis such as HIV infection, malignancies, cirrhosis, organ transplantation, end-stage renal failure, autoimmune disorder, diabetes mellitus, idiopathic CD4 T-cell lymphopenia, sarcoidosis, chronic usage of corticosteroids or other immunosuppressive therapies, and any abnormal symptoms were regarded as patients “without apparent risk factors” (*2*). Since 2002, the period when isolates were obtained from 68% of the patients without apparent risk factors, HIV serologic testing and a battery of immunologic tests were performed on all cryptococcosis patients. Before 2002, each patient’s cellular and humoral immune status was routinely determined. Any abnormality in these tests led to further HIV serology. The patients who had otherwise normal test results were also subjected to HIV serologic testing, the results of which were negative.

**Figure 1:**
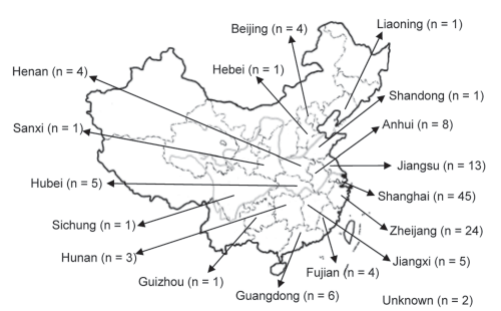
Mainland China. The numbers in the boxes represent strains used in this study that were isolated during 1980–2006 from each region.

**Figure 2:**
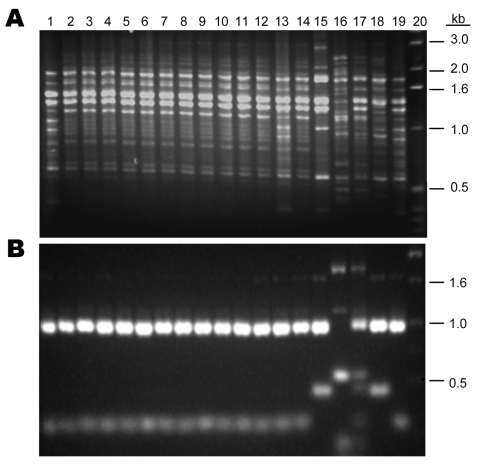
DNA fingerprint patterns of 12 *Cryptococcus neoformans* strains from China and the molecular type reference strains. A) M13-based PCR pattern. B) *URA5* restriction fragment length polymorphism. Lanes: 1, VNI; 2–12, 11 Chinese strains; 13, H99; 14, Chinese strain B-4587; 15, VNBt63; 16, VNI; 17, VNIII; 18, VNII; 19, VNI; 20, marker.

Of the 120 *C. neoformans* strains obtained from 16 provinces located in the middle to the eastern regions of mainland China (Figure 1), 84 (70 %) strains were isolated from apparently healthy patients and 27 (22.5 %) strains were isolated from patients with risk factors other than HIV infection (Appendix Table). Only 9 (7.5 %) of the *C. neoformans* strains were isolated from AIDS patients. All 120 isolates were analyzed by PCR fingerprints using M13 primers, by *URA5* RFLP patterns, and by (GACA)_4_ sequence primers. Notably, all 120 strains of *C. neoformans* yielded an identical M13-based fingerprint pattern that could be distinguished from the reference types. In Figure 2, the M13 fingerprint patterns of 12 of the 120 strains are shown as examples along with the 6 reference strains. The major bands pattern for the serotype A isolates from China was more similar to the VNI than the other reference types (Figure 2, panel** A**). The *URA5* RFLP (Figure 2, panel** B**) and (GACA)_4_ patterns of 120 isolates, however, were identical to that of the VNI type (Appendix Figure 1).

**Figure 3:**
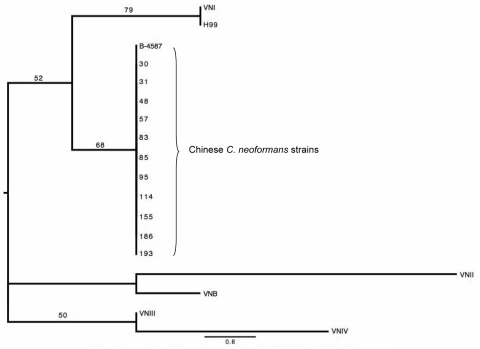
The phylogenetic tree for maximum parsimony analysis composed on the basis of the M13-PCR pattern of 12 Chinese *Cryptococcus neoformans* strains. Numbers above the branches represent bootstrap support percentages based on 500 replicates. The scale bar represents the inferred number of steps along a branch of the tree.

The phylogenetic tree for maximum parsimony analysis showed the strains to be closely related to the reference strain VNI and the H99 strain (Figure 3). The H99 strain had the same M13 fingerprint pattern as that of the VNI strain. Genotyping by sequence analysis of the IGSI- rDNA region indicated that the *C. neoformans* strains from China belonged to the *C. neoformans* genotype 1a (Appendix Figure 2). Genotype 1a is the major genotype found among the serotype A strains of *C. neoformans* collected worldwide and follows a clonal pattern (*9*).

**Figure 4:**
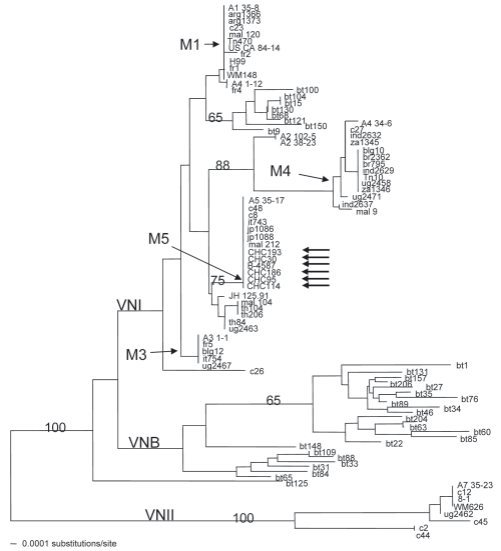
Genetic relationship of multilocus sequence typing (MLST) genotypes among 94 isolates of *Cryptococcus neoformans* serotype A (88 strains from Litvintseva et al. [*10*]) and 6 representative Chinese strains) visualized by the neighbor-joining dendrogram. Numbers on each branch indicate the bootstrap values >50%, based on 500 replicates. Vertical lines represent strains with identical genotypes. Arrows indicate MLST results for Chinese strains.

**Figure 5:**
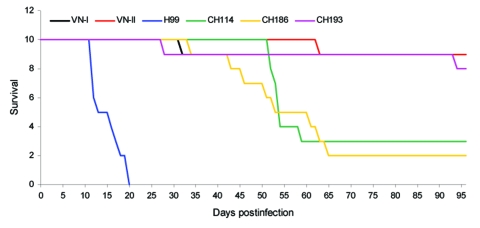
Virulence in mice. Mice were intranasally infected with 5 × 10^7^ yeast cells from the 3 Chinese *Cryptococcus neoformans* strains, CHC114, CHC193, and CHC 186, and compared with H99 and the reference strains VNI and VNII for survival.

MLST, performed using 8 unlinked genes from 6 randomly chosen strains, showed identical sequences for the *CAP10, GPD, LAC1, PLB1, SOD1, IGSI, TEF1*, and *URE1* genes. These results corroborate the homogeneity observed with various PCR fingerprint patterns. Notably, the Chinese strains of *C. neoformans* formed a cluster with 7 strains previously reported by Litvintseva et al. (Table) (*10*) and formed a distinct cluster, M5, that appeared to have diverged from the M1 genotype to which the VNI reference strain belongs (Figure 4). Two strains in the M5 that clustered with the Chinese strains, jp1086 and jp1088, were isolated in Japan and the M-13 fingerprint pattern of all 7 strains (Table) tested was identical to that of the Chinese strains (Appendix Figure 3). Our genotyping data indicates that M13-based PCR fingerprinting together with the MLST are powerful tools that enable discrimination of different strain types. The 3 Chinese *C. neoformans* strains randomly chosen to assess virulence were considerably less virulent than the H99 strain and moderately to significantly more virulent than VNI and VNII reference strains (Figure 5).

**Table:** Information on 7 *Cryptococcus neoformans* strains previously determined as M5 of MLST*

Strain	Geographic origin	Source	MAT	Year	MLST type	VN type	Reference
Jt743	Italy	Unknown	*α*	Unknown	M5	VNI	S. Maesaki
Jp1086	Japan	Human lung	*α*	1999	M5	VNI	S. Maesaki
Jp1088	Japan	Human lung	*α*	1999	M5	VNI	(*10*)
mal 212 i	Malawi	CSF	*α*	1999	Μ5	VNI	(*25*)
C48	USA	Bronchial lavage	*α*	2001	Μ5	VNI	(*10*)
C8	USA	CSF	*α*	2001	Μ5	VNI	(*10*)
A5 36–17	USA	Pigeon excreta	*α*	2002	Μ5	VNI	(*10*)

*MLST, multilocus sequence typing; MAT, mating type; CSF, cerebrospinal fluid.

The H99 strain, the type strain of *C. neoformans* var. *grubii* (*26*), clustered with VNI strain based on the MLST data. The previous MLST tree, which did not place the H99 strain in the same cluster, A1 + A3 (= M1), as VNI was determined to be due to an error introduced during the sequencing process (*10*). The sequences from 8 genes that we sequenced matched 100% with the nucleotide sequence data posted in the H99 genomic database.

**Figure 6:**
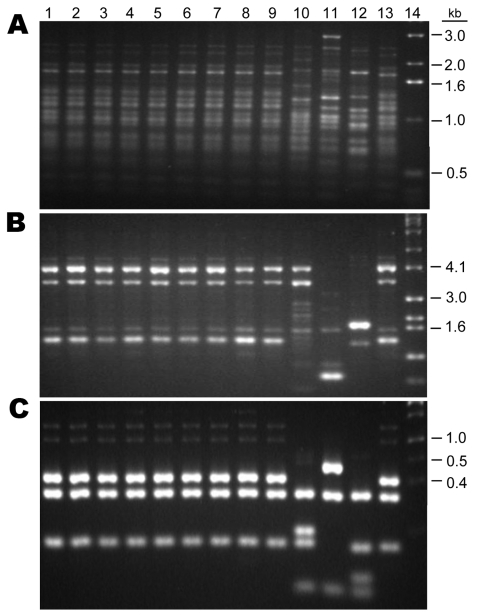
Comparison of the PCR patterns of 9 Chinese *Cryptococcus gattii* isolates with reference *C. gattii* strains. A) M13, B) (GACA)_4_, C) URA5. Lanes: 1–9, Chinese strains; 10, VGIV; 11, VGIII; 12, VGII; 13, VGI; 14, marker.

Nine isolates of *C. gattii* were all obtained from the eastern regions of China. Except for 1 strain from Shangdong, and 1 from Shanghai, all strains were recovered from provinces located south of Shanghai with a warm climate. Five (55%) of the 9 strains of *C. gattii* were from the Zhejiang province where *Eucalyptus* trees are commonly found. Of the 9 *C. gattii* strains, 2 were isolated from AIDS patients and 7 were isolated from otherwise normal patients. All of the *C. gattii* strains were serotype B, of *MATα* mating type with typical PCR patterns of the VGI type (Figure 6). IGS sequences from 4 randomly chosen strains belonged to the genotype 4b (*9*).

## Discussion

From 1985 to 2007, a total of 7,372 cases of cryptococcosis were reported in China. However, documentation on these patients is mostly unavailable, and fragmented documentation is available for only 1,999 cases. Among them, 323 (16.2%) cases seem to have occurred in patients with no underlying disease and who were considered otherwise healthy; 215 (10.8%) cases occurred in AIDS patients (Z. Yao et al., unpub. data).

This study provides a large scale population analysis of *C. neoformans* strains isolated from 129 patients with well-documented clinical cases of cryptococcosis that were treated at the Shanhai Changzheng Hospital. All patients were assessed for the common predisposing factors for cryptococcosis. Patients without any of these predisposing factors were regarded as “with no apparent risk factor.” HIV serology was performed for all cases that have occurred since 2002. A total of 68% (56/82) of the patients had no underlying disease. Patient outcomes were monitored for 2 years following treatment at which time patients were determined to be cured of cryptococcosis.

Consistent with previous reports, all 120 clinical strains of *C. neoformans* isolated from China were serotype A and *MATα* (*2*). In contrast to the genotypic diversity of clinical *C. neoformans* serotype A strains found in other countries, such as Brazil, Australia, and the United States (*5*,*27*), the Chinese *C. neoformans* strains showed remarkable genetic homogeneity. This was evident not only in the patterns based on various PCR fingerprints but also by the lack of diversity in MLST results. A report from India also showed relatively low genetic diversity among 57 clinical *C. neoformans* isolates. However, a serotype D strain was found, indicating that the population structure of *C. neoformans* in India is less homogeneous than that in China (*28*). Remarkably, a majority of the Chinese strains caused cryptococcosis in persons without any recognizable immune defect or underlying disease. Although a considerable number of AIDS patients have been identified, 8.5% of the 129 strains isolated across mainland China were recovered from AIDS patients; 71% were isolated from otherwise healthy persons. This is in stark contrast to the >80% AIDS-associated cryptococcosis cases reported in Europe and the United States and the 69% reported in Africa (*29*–*31*). Although markedly lower than the frequency in China, a relatively high number of cases of non-AIDS associated cryptococcosis due to *C. neoformans* were documented in Australia, New Zealand (*32*), and India (*28*). Most patients in Australia and New Zealand, however, were immunocompromised (*32*). The report from India showed that 41% of the cryptococcosis patients also had HIV infections; the remaining patients were determined to have no known immune defect (*28*). Information on the underlying diseases in these HIV-free Indian patients, however, was not recorded.

We recognize the possibility that 71% of the Chinese cryptococcosis patients without apparent underlying disease may have had subtle defects in immunity that may have predisposed them to cryptococcosis. Alternatively, genetic factors may play an important role in the unusually high non–AIDS-associated cryptococcosis in China. A relationship between common functional genetic polymorphisms of the low-affinity Fc gamma receptor genes, FCGR2A, -3A, and -3B, and the risk of cryptococcosis in HIV-uninfected patients was recently reported (*33*). It would be of interest to investigate the relationship between the genetic polymorphisms of the 3 genes in the immunocompetent Chinese cryptococcocis patients.

The M13 based PCR fingerprints of the Chinese *C. neoformans* strains were identical to each other and similar but distinguishable from VNI. For convenience throughout the discussion, we will hereafter refer to the M13 pattern of the Chinese strains as VNIc. The MLST-based phylogenetic tree also showed that the VNIc diverged from WM148 and formed a separate cluster with 7 previously analyzed strains (*10*). The 7 strains that cluster with the VNIc strains had originated from 3 different continents, which suggests that the VNIc type is not unique to China and apparently follows a widespread cosmopolitan distribution. We found that the 7 strains that cluster with Chinese strains have exactly the same M13 PCR pattern as the VNIc type. Whether most of these 8 strains were also from immunocompetent patients is not known. Since analysis of the IGSI region of the VNIc strains showed it to be identical with WM148, the M13 fingerprint analysis appears to have greater discriminatory power in distinguishing the VNIc strains from the typical VNI type than IGSI analysis. The *URA5* RFLP analysis or the (GACA)_4_-based PCR fingerprints also had lower resolution power than the M13 pattern analysis because the 2 methods could not distinguish VNIc from the WM148 strain.

We do not know if VNIc is the most common type in China because the present study only analyzed clinical strains. Environmental strains should also be isolated and undergo molecular typing. Such a study would offer insight into the importance of the VNIc strain type in relation to infection in otherwise healthy persons in China. In general, the occurrence of cryptococcosis in patients without any apparent immune defect or underlying disease is relatively rare (*2*,*34*). In the United States, the incidence of cryptococcosis in immunocompetent patients has been estimated at 0.2/1,000,000 population per year in California (*35*); 0.9/100,000 in Atlanta, Georgia; and 0.93/100,000 in Alabama (*36*).

The presence of the VGI type in China is not surprising because it is the most common *C. gattii* strain type identified in Southeast Asia and most of the *C. gattii* strains obtained were from the southeastern part of China. Although the total number of the *C. gattii* strains studied is small, the percentage of AIDS-associated *C. gattii* infections in China is higher than expected. Other than from certain geographic areas in Africa (*4*), *C. gattii* strains are rarely isolated from AIDS patients (*2*,*32*). In Australia where *C. gattii* is prevalent, for example, only 1 of 47 clinical strains of *C. gattii* was isolated from a patient with AIDS (*32*).

In our previous study, the urease-negative strain B-4578, which had been isolated from a cryptococcosis patient in China, was as virulent in mice as was the highly virulent H99 strain (*23*). Because the strain B-4578 was also of the VNIc molecular type, it was tempting to assume that strains isolated from immunocompetent patients in China would similarly be highly virulent in experimental animals. Indeed, the 3 strains of the VNIc type isolated from different provinces tested in mice were more virulent than the VNI reference strain. However, they were considerably less virulent than the H99 strain, which had been isolated from an immunocompromised patient (*20*). This suggests that a wide variation in virulence exists among the strains of the VNIc molecular type. Whether the degree of cryptococcal strain virulence manifested in mice is comparable to the human host remains unknown. Our experience with strain NIH12 has shown that virulence in mice does not necessarily correlate with that of the human host. The strain NIH12 is one of the most virulent serotype D strains tested in BALB/c mice (*37*); it only caused a chronic localized infection without dissemination in the human host. The patient infected by the NIH12 strain had sarcoidosis and a chronic, localized, osteomyelitis lesion later developed in the hip, which was cured by amphotericin B treatment without any dissemination or recurrence.

Because all 129 of the cryptococcal strains were isolated from Chinese, primarily immunocompetent, patients, one can ask whether any susceptibility difference to cryptococcosis is related to ethnicity. Australian studies have indicated a higher frequency of cryptococcosis in Aborigines (*38*) than in whites, and in Los Angeles, disease incidence was reported to be twice as frequent in Hispanics than in whites (*39*). However, data regarding ethnic differences in susceptibilities are scant and unconvincing. Since most of the 129 strains were isolated from non-AIDS patients in China, possible differences may exist in the reporting systems for cryptococcosis cases among AIDS patients and among non-AIDS patients. While some strains were isolated from patients in Henen, Yunan, and Xinjian provinces where HIV/AIDS was more prevalent, most strains were from patients in regions where HIV/AIDS was not prevalent. Although the HIV status of some otherwise apparently healthy patients was unknown, they did fully recover after antifungal treatment. The outcome of the AIDS patients with cryptococcosis, however, is unknown because they were transferred to a quarantined facility soon after they were diagnosed to be HIV positive. To determine whether Chinese AIDS patients are more resistant to cryptococcosis than AIDS patients in other countries, differences in how AIDS patients are handled in China should be investigated.

In conclusion, this study reveals a strikingly homogeneous cryptococcal population belonging to a subtype of VNI in China. The high incidence of cryptococcosis in immunocompetent patients in China contrasts with reports from other countries.

## Supplementary Material

Appendix Table Cryptococcal isolates used in this study

Appendix Figure 1 (GACA)4 comparison between fingerprint pattern of Chinese Cryptococcus neoformans strains and reference strains. Lanes: 1, VNI; 2-12, 11 Chinese strains; 13, H99; 14, Chinese strain B-4587; 15, VNBt63; 16, VNI; 17, VNIII; 18, VNII; 19, VNI; 20, marker.

Appendix Figure 2 Phylogenetic tree constructed on the partial sequence of intergenic spacer region 1 (IGS)1-5.8S-IGSII region. The tree was computed with PAUP*4 (24) (heuristic search, stepwise addition, random addition sequence, nearest neighbor interchange, 100 maximum trees). Numbers represent bootstrap values of 500 replicates. Sequence data contained a total of 770 characters (747 constant characters; 4 uninformative characters; 19 parsimony informative characters). Gaps were represented as missing data. Each character was treated as an independent, unordered, multiple character of equal weight.

Appendix Figure 3 M13-PCR fingerprint pattern of the 7 strains in the M5 cluster (lanes 5-9), a Chinese strain (CHC123, lane 2), and VNI (lane 1).
