# Supplementary material for: Cryptococcus neoformans Strains and Infection in Apparently Immunocompetent Patients, China
Source: Emerg Infect Dis. 2008 May;14(5):755–62. doi: 10.3201/eid1405.071312 (PMC2600263; doi:10.3201/eid1405.071312)
Supplement: Appendix Table — Cryptococcal isolates used in this study [file 07-1312_appT-s1.pdf]

Appendix Table. Cryptococcal isolates used in this study

Isolate	Patient sex	Source of isolation	Geographic origin	Underlying disease	Serotype	Mating type	Year of isolation
CHC-1	F	CSF	Henan	AIDS	A	α	2004
CHC-2	M	CSF	Shanghai	None	A	α	2005
CHC-3	M	CSF	Shanghai	None	A	α	2005
CHC-4	M	CSF	Shanghai	None	A	α	2000
CHC-5	F	CSF	Anhui	None	A	α	2006
CHC-6	F	CSF	Jiangsu	None	A	α	2006
CHC-7	F	CSF	Shanghai	None	A	α	2004
CHC-8	M	CSF	Jiangxi	SLE	A	α	2006
CHC-9	M	CSF	Jiangsu	Hepatic enecephalopathy	A	α	2004
CHC-12	F	CSF	Zhejiang	None	A	α	2005
CHC-13	F	Cisternal fluid	Zhejiang	None	A	α	2004
CHC-15	F	CSF	Zhejiang	None	B	α	2004
CHC-16	M	CSF	Zhejiang	None	B	α	2004
CHC-17	M	CSF	Unknown	None	A	α	1996
CHC-18	M	CSF	Shanghai	None	A	α	2005
CHC-19	M	CSF	Liaoning	Hemolytic anemia, diabetes	A	α	2004
CHC-21	M	Bronchial lavage	Shanghai	None	A	α	2004
CHC-22	F	CSF	Shanghai	AIDS	A	α	2006
CHC-24	F	CSF	Guangdong	None	B	α	2006
CHC-25	F	CSF	Zhejiang	Liver fibrosis	A	α	2006
CHC-26	F	Bronchial lavage	Shanghai	None	A	α	2006
CHC-28	F	Sputum	Shanghai	SLE	A	α	2006
CHC-29	M	CSF	Zhejiang	None	A	α	2006
CHC-30	M	CSF	Jiangxi	None	A	α	2006
CHC-31	M	CSF	Hubei	None	A	α	2006
CHC-32	F	CSF	Hunan	None	A	α	2003
CHC-33	M	CSF	Hubei	None	A	α	2004
CHC-34	M	CSF	Anhui	None	A	α	2004
CHC-35	M	CSF	Guangdong	None	A	α	2006
CHC-36	M	CSF	Unknown	None	A	α	2006
CHC-37	M	CSF	Zhejiang	None	A	α	1996
CHC-38	M	CSF	Zhejiang	None	A	α	1997
CHC-40	F	CSF	Zhejiang	None	B	α	2003
CHC-42	M	CSF	Shanghai	SLE	A	α	2003
CHC-43	M	CSF	Shanghai	None	A	α	2005
CHC-44	M	Bronchial lavage	Anhui	Liver cancer, diabetes	A	α	2004
CHC-48	F	CSF	Shanghai	None	A	α	1995
CHC-57	M	CSF	Henan	None	A	α	2003
CHC-75	F	CSF	Henan	None	A	α	2003
CHC-76	F	CSF	Shanghai	None	A	α	2003
CHC-83	F	CSF	Guangdong	None	A	α	2001
CHC-84	M	CSF	Guangdong	None	A	α	2000
CHC-85	M	CSF	Sanxi	None	A	α	1996
CHC-89	M	CSF	Zhejiang	None	A	α	1993
CHC-95	F	CSF	Anhui	None	A	α	2000
CHC-98	F	Stool	Shanghai	Chronic granulocytic leukemia	A	α	2006
CHC-100	F	Stool	Shanghai	Idiopathic CD+4 cell lymphocytopenia	A	α	1998
CHC-102	M	CSF	Guangdong	None	A	α	2006
CHC-105	M	CSF	Henan	None	A	α	2000
CHC-112	M	CSF	Shanghai	None	A	α	2001
CHC-113	M	CSF	Shanghai	None	A	α	2000
CHC-114	F	CSF	Hebei	None	A	α	1999
CHC-118	F	Bronchial lavage	Shanghai	None	A	α	2002
CHC-120	M	CSF	Anhui	Chemotherapy postcancer surgery	A	α	1999
CHC-123	M	CSF	Beijing	AIDS	A	α	2006
CHC-124	M	CSF	Beijing	AIDS	A	α	2006
CHC-125	M	CSF	Beijing	AIDS	A	α	2006
CHC-126	M	CSF	Beijing	AIDS	A	α	2006
CHC-128	M	CSF	Hubei	None	A	α	2006
CHC-132	F	Sputum	Hunan	Kidney transplant	A	α	2002
CHC-133	M	CSF	Jiangsu	None	A	α	1999
CHC-134	M	CSF	Jiangxi	Kidney transplant	A	α	2002
CHC-135	F	Sputum	Zhejiang	None	A	α	2004

CHC-136	M	CSF	Hubei	None	A	α	2002
CHC-137	M	CSF	Jiangsu	None	A	α	2001
CHC-138	M	CSF	Zhejiang	None	A	α	2005
CHC-139	M	CSF	Hubei	None	A	α	2006
CHC-140	M	Blood	Zhejiang	Kidney failure	A	α	2003
CHC-141	F	CSF	Sichung	SLE	A	α	2006
CHC-142	M	CSF	Fujian	None	A	α	2000
CHC-143	M	CSF	Jiangsu	None	A	α	2002
CHC-144	M	CSF	Jiangsu	None	A	α	2003
CHC-145	M	CSF	Guangdong	None	A	α	2006
CHC-146	M	CSF	Shanghai	SLE	A	α	2000
CHC-147	M	CSF	Zhejiang	None	A	α	2002
CHC-148	F	Sputum	Shanghai	AIDS	A	α	2002
CHC-149	F	CSF	Shanghai	SLE	A	α	2005
CHC-150	F	CSF	Shanghai	None	A	α	2002
CHC-151	M	CSF	Anhui	None	A	α	2002
CHC-152	M	CSF	Anhui	None	A	α	2006
CHC-153	M	CSF	Shanghai	Chronic granulocytic leukemia	A	α	2000
CHC-154	M	CSF	Shandong	AIDS	B	α	1995
CHC-155	M	CSF	Jiangsu	None	A	α	2006
CHC-156	M	CSF	Shanghai	AIDS	A	α	2006
CHC-157	M	CSF	Shanghai	None	A	α	2002
CHC-158	M	CSF	Zhejiang	None	A	α	2001
CHC-159	M	CSF	Zhejiang	None	A	α	2006
CHC-160	M	CSF	Zhejiang	Liver fibrosis	A	α	2001
CHC-161	F	CSF	Shanghai	SLE	A	α	2006
CHC-162	F	CSF	Jiangxi	SLE	A	α	2000
CHC-163	F	CSF	Jiangxi	SLE	A	α	2000
CHC-164	F	CSF	Shanghai	None	A	α	2006
CHC-172	F	CSF	Shanghai	AIDS	A	α	2004
CHC-174	F	CSF	Jiangsu	Chemotherapy postcancer surgery	A	α	1997
CHC-175	M	CSF	Anhui	Chemotherapy postcancer surgery	A	α	1999
CHC-176	F	CSF	Zhejiang	None	A	α	1997
CHC-177	F	CSF	Shanghai	SLE	A	α	1998
CHC-178	M	CSF	Zhejiang	None	A	α	1993
CHC-179	M	CSF	Jiangsu	None	A	α	2005
CHC-180	F	CSF	Shanghai	None	A	α	1994
CHC-181	M	CSF	Shanghai	None	A	α	1994
CHC-182	F	CSF	Shanghai	None	A	α	1994
CHC-183	F	CSF	Shanghai	None	A	α	2005
CHC-185	M	CSF	Shanghai	SLE	A	α	2003
CHC-186	M	CSF	Hunan	None	A	α	2001
CHC-187	M	CSF	Shanghai	None	A	α	2003
CHC-188	M	CSF	Shanghai	None	A	α	2002
CHC-189	M	CSF	Zhejiang	None	A	α	2000
CHC-190	M	CSF	Zhejiang	None	A	α	2003
CHC-191	M	CSF	Shanghai	None	A	α	1993
CHC-192	M	CSF	Shanghai	None	A	α	1994
CHC-193	M	CSF	Fujian	None	A	α	1998
CHC-194	F	CSF	Fujian	None	A	α	1999
CHC-195	F	CSF	Shanghai	None	A	α	1992
CHC-196	F	CSF	Shanghai	None	A	α	1995
CHC-197	M	CSF	Shanghai	None	A	α	1990
CHC-198	M	CSF	Jiangsu	None	A	α	2003
CHC-199	F	CSF	Jiangsu	None	A	α	1995
CHC-200	F	CSF	Zhejiang	None	A	α	1994
CHC-201	M	CSF	Zhejiang	None	B	α	1996
CHC-202	M	CSF	Jiangsu	None	A	α	1995
CHC-203	F	CSF	Jiangsu	None	B	α	1994
CHC-204	F	CSF	Shanghai	SLE	A	α	2003
CHC-205	F	Sputum	Shanghai	Idiopathic CD+4 cell lymphocytopenia	A	α	2000
CHC-208	M	CSF	Fujian	None	A	α	2003
CHC-209	M	CSF	Shanghai	AIDS	B	α	2005
CHC-210	F	CSF	Shanghai	None	A	α	1980
B4587	M	CSF	Guizhou	None	A	α	1998

*CSF, cerebrospinal fluid; SLE, systemic lupus erythrematosus.
