# Supplementary figures and images for: Cryptococcus neoformans Strains and Infection in Apparently Immunocompetent Patients, China
Source: Emerg Infect Dis. 2008 May;14(5):755–62. doi: 10.3201/eid1405.071312 (PMC2600263; doi:10.3201/eid1405.071312)

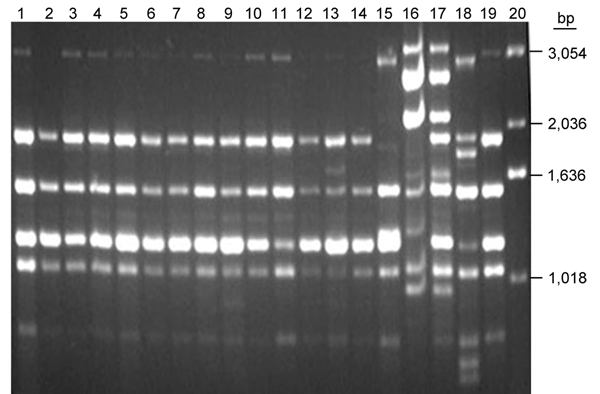

Supplement: Appendix Figure 1 — (GACA)4 comparison between fingerprint pattern of Chinese Cryptococcus neoformans strains and reference strains. Lanes: 1, VNI; 2-12, 11 Chinese strains; 13, H99; 14, Chinese strain B-4587; 15, VNBt63; 16, VNI; 17, VNIII; 18, VNII; 19, VNI; 20, marker. [file 07-1312_app1-s2.gif]

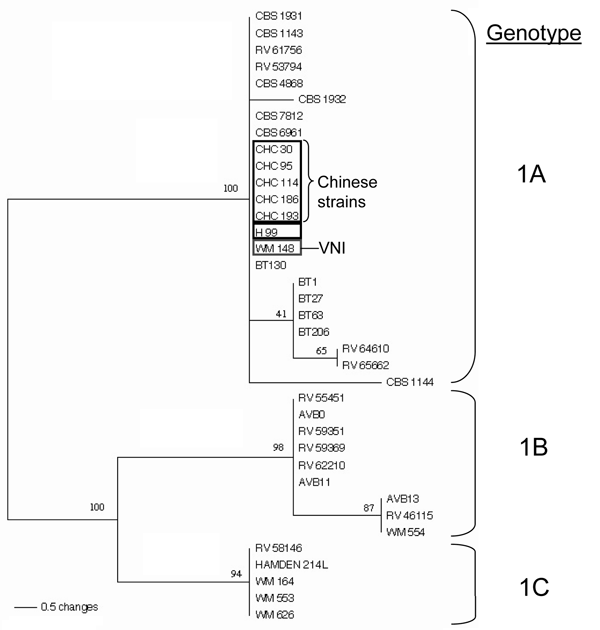

Supplement: Appendix Figure 2 — Phylogenetic tree constructed on the partial sequence of intergenic spacer region 1 (IGS)1-5.8S-IGSII region. The tree was computed with PAUP*4 (24) (heuristic search, stepwise addition, random addition sequence, nearest neighbor interchange, 100 maximum trees). Numbers represent bootstrap values of 500 replicates. Sequence data contained a total of 770 characters (747 constant characters; 4 uninformative characters; 19 parsimony informative characters). Gaps were represented as missing data. Each character was treated as an independent, unordered, multiple character of equal weight. [file 07-1312_app2-s3.gif]

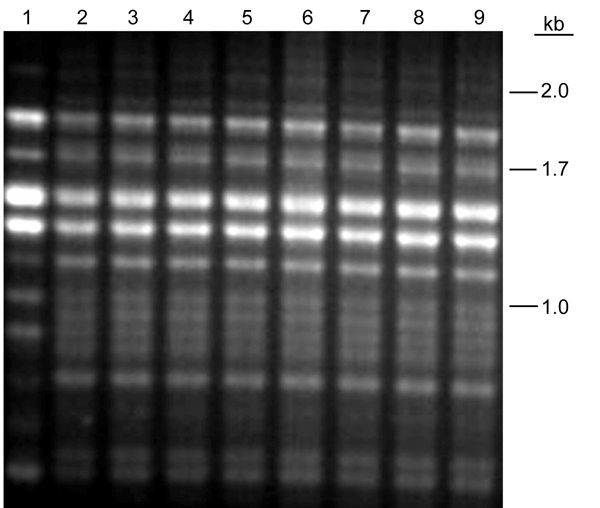

Supplement: Appendix Figure 3 — M13-PCR fingerprint pattern of the 7 strains in the M5 cluster (lanes 5-9), a Chinese strain (CHC123, lane 2), and VNI (lane 1). [file 07-1312_app3-s4.gif]
